# Comparing population health in the United States and Canada

**DOI:** 10.1186/1478-7954-8-8

**Published:** 2010-04-29

**Authors:** David Feeny, Mark S Kaplan, Nathalie Huguet, Bentson H McFarland

**Affiliations:** 1 The Center for Health Research, Kaiser Permanente Northwest, 3800 N Interstate Avenue, Portland, OR, 97227, USA; 2 University of Alberta and Institute of Health Economics, 10405 Jasper Avenue, #1200, Edmonton, AB, T5J 3N4, Canada; 3 Health Utilities Incorporated, 88 Sydenham Street, Dundas, ON, L9H 2V3, Canada; 4 Department of Community Health, Portland State University, 506 SW Mill Street, Portland, OR, 97201, USA; 5 Research Associate, Center for Public Health Studies, Portland State University, 506 SW Mill Street, Portland, OR, 97201, USA; 6 Departments of Psychiatry and Public Health and Preventive Medicine, Oregon Health & Science University, 3181 SW Sam Jackson Park Road, Portland, OR, 97239, USA

## Abstract

**Background:**

The objective of the paper is to compare population health in the United States (US) and Canada. Although the two countries are very similar in many ways, there are potentially important differences in the levels of social and economic inequality and the organization and financing of and access to health care in the two countries.

**Methods:**

Data are from the Joint Canada/United States Survey of Health 2002/03. The Health Utilities Index Mark 3 (HUI3) was used to measure overall health-related quality of life (HRQL). Mean HUI3 scores were compared, adjusting for major determinants of health, including body mass index, smoking, education, gender, race, and income. In addition, estimates of life expectancy were compared. Finally, mean HUI3 scores by age and gender and Canadian and US life tables were used to estimate health-adjusted life expectancy (HALE).

**Results:**

Life expectancy in Canada is higher than in the US. For those < 40 years, there were no differences in HRQL between the US and Canada. For the 40+ group, HRQL appears to be higher in Canada. The results comparing the white-only population in both countries were very similar. For a 19-year-old, HALE was 52.0 years in Canada and 49.3 in the US.

**Conclusions:**

The population of Canada appears to be substantially healthier than the US population with respect to life expectancy, HRQL, and HALE. Factors that account for the difference may include access to health care over the full life span (universal health insurance) and lower levels of social and economic inequality, especially among the elderly.

## Background

Canada and the United States (US) share a common border and enjoy very similar standards of living. Training standards for health care professionals are also very similar. Yet, the two societies differ in important ways. First, Canada provides universal first-dollar (i.e., no co-pay) health care insurance coverage for medical and hospital services. In the US, access to health insurance is typically based on employment, income (Medicaid), or age (Medicare), is not universal, and varies in coverage and co-pay requirement. Second, health care expenditures have been higher in the US than in Canada since the early 1970s [1-3]. Third, the degree of social and economic inequality is higher in the US [1,3,4].

Do these differences in the organization of health care and inequality have implications for the health of the two populations? A recent health survey (Joint Canada/US Health Survey, Statistics Canada, and National Center for Health Statistics, JCUSH) provides data on health status and health-related quality of life (HRQL) to address these questions. The objectives of this paper are to compare HRQL, life expectancy, and health-adjusted life expectancy (HALE) [5-10] in the two countries. HALE provides a comprehensive comparison of population health in the two countries, taking into account both mortality and morbidity.

## Methods

Health in the US and Canada are compared in three different ways. First, we compare mean HRQL scores by age. It is well-known that health is affected by more than health care [11-13]. Because the distribution of risk factors differs between the two countries, comparisons will be based on results adjusted for standard risk factors and determinants of health variables [11]: gender, race, education, income, smoking status, and body mass index (BMI). Further, given that the legacies of slavery and racial discrimination in the US affect population health, comparisons of the white-only Canadian and US populations are included [14-17]. Second, we compare estimates of life expectancy to take into account differing mortality rates between the two countries. Finally, HALE estimates provide a comprehensive comparison, taking into account differences in both morbidity and mortality. To put the results from the JCUSH into context, the demographic results reported in the JCUSH for the US and Canada are compared to contemporary population health surveys of the non-institutionalized population in each country and comparisons of the prevalence of major chronic conditions based on data from the JCUSH in the two countries are provided descriptively.

### Data Sources

The data are derived from the 2002/03 JCUSH, a telephone-interview population health survey conducted jointly by Statistics Canada and the US National Center for Health Statistics [18-20]. All JCUSH interviews were conducted by the regional offices of Statistics Canada. The content of the JCUSH was developed using questionnaire items from the Canadian Community Health Survey and the US National Health Interview Survey. The JCUSH is the first survey to provide fully comparable data of health status, lifestyle, health care utilization, and other determinants of health. Using stratified random sampling and random digit-dialing procedures, the JCUSH interviewed 3,505 Canadian and 5,183 American non-institutionalized persons aged 18 and older. The data accessed for the analyses reported here were taken from a public-use data file designed to ensure the confidentiality of subjects.

### Measures

The Health Utilities Index Mark 3 (HUI3) was used to measure HRQL. HUI3 is based on eight dimensions, or attributes, of health status: vision, hearing, speech, ambulation dexterity, emotion, cognition, and pain and discomfort [21-24]. Each attribute has five or six levels, ranging from normal to severely disabled. An individual's health status at a time point is described by an eight-element vector consisting of one level for each attribute. Overall HUI3 scores derive from a multiplicative, multi-attribute utility function based on preference scores from a random sample of the Canadian population. Overall HUI3 scores are on the conventional scale in which dead = 0.00 and perfect health = 1.00.

There is extensive evidence on the construct validity of HUI3 in assessing population health and the burden associated with various chronic conditions. including Alzheimer's disease, arthritis, cataracts, diabetes, stroke, obesity, Parkinson's disease, and multiple sclerosis [5,22,25-34]. Evidence of the ability of baseline HUI3 scores to predict subsequent mortality, controlling for standard determinants of health, is found in Kaplan and colleagues [35] and Wilkins [36], providing evidence of the predictive validity of HUI3.

Differences of 0.03 or more in overall HUI3 scores are regarded as clearly important [22,25,37]; differences as small as 0.01 may be important, especially in the context of population health [37]. The 0.03 guideline is based on evidence from cross-sectional and longitudinal known-groups comparisons and inspection of the HUI3 scoring function. Changes of one level on any attribute in the HUI3 system are regarded as clinically important and imply, in general, a change of 0.03 or more in overall HUI3 score. In population health studies, the decrement in overall HUI3 scores associated with major chronic conditions is, in general, ≥0.03 [23-29]. Using data from a major Canadian population health survey, the National Population Health Survey (NPHS), Trakas et al. [32] report differences in overall HUI3 scores of 0.02 when comparing the normal and overweight group (BMI 19.0 - 29.9) to the obese Class I group (BMI 30.0 - 34.9) and Class I to obese Class II (BMI ≥ 35.0). McIntosh et al. [38], using data from the 2000-2001 Canadian Community Health Survey, report differences in mean HUI3 score for males by education of 0.02 comparing postsecondary diploma to secondary graduation and 0.012 comparing university degree to postsecondary diploma. For females, the corresponding differences are 0.021 and 0.026. Thus, in known-group comparisons in population health survey data, important differences as small as 0.01 and 0.02 in mean HUI3 scores are observed.

### Statistical analyses

We used multiple linear regression to compute the adjusted HUI3 means for each age in both samples. Analyses were weighted to reflect the sample design, and standard errors and significance tests were adjusted for nonresponse and post-stratification [18] using SUDAAN (Release 9.0.1; Research Triangle Institute, Research Triangle Park, NC).

#### Computation of life expectancy and health-adjusted life expectancy in Canada and the United States

In addition to comparing HRQL and life expectancy in the two countries, we estimated HALE to reflect differences in both mortality and morbidity more comprehensively. The computation of HALE is based on the Sullivan method that combines age-specific mortality rates and age-specific utility scores to assess the HRQL of survival in that age range [39].

Age-specific death rates for 2003 were obtained from Canadian Vital Statistics [40,41] and the US National Vital Statistics Report [42]. Average annual death rates for five-year age groups were then computed for 13 age intervals beginning at age 20 and ending with age 84 (i.e., 20-24, 25-29,..., 80-84). Regarding the 14^th ^and oldest possible age interval, it was assumed that all individuals alive at age 85 died before age 95. It was also assumed that people who died during an age interval expired at the mid-point of the interval.

The average HUI3 score for each five-year age group was obtained from the JCUSH. Owing to small numbers of respondents over age 84, the average HUI3 score for the 85-94 age group was set initially at 0.6 for both Canada and the US. This value is roughly 0.1 below the average HUI3 score in the 80-84 age group. This assumption is addressed subsequently.

Within each age interval, the health-adjusted life years were computed by multiplying the average HUI3 score for the age group by the number of years in the age interval (five years for the 13 age intervals prior to age 85 and 10 years for the 85-94 age interval). The health-adjusted life years were then summed to yield the cumulative health-adjusted life years to the mid-point of each age interval.

The assumption that the average HUI3 score in the 85-94 age group is 0.6 was addressed via sensitivity analysis. The average HUI3 score in the 85-94 age group was varied by 0.1 unit from 0.1 through 0.7, the corresponding Canadian and US health-adjusted life expectancies at age 19 were calculated, and the differences (Canada minus US) were computed. The differences were 2.70, 2.74, 2.78, 2.82, 2.86, 2.90, and 2.94 years of health-adjusted life, respectively. In other words, varying the assumed HUI3 score in the 84-94 age group over its range had minimal impact (0.2 years, or about two months of health-adjusted life) on the Canada-versus-US differences.

Of course, age-specific mortality rates and age-specific mean HUI3 scores are measured with imprecision. The most important limitation is the modest sample size of the JCUSH used as the source for the mean HUI3 scores for each age group. In order to assess the degree of precision in the estimates of HALE and to assess the impact of the modest sample size of the JCUSH, we have assumed that mortality rates are measured without error. (Evidence on the degree of precision of Canadian mortality rates is found in [43].) We have used the standard errors for the age-specific mean HUI3 scores to assess the degree of precision of the estimates of HALE for each country and the statistical significance of differences in the estimates of HALE.

Counterfactual estimates were then computed in order to decompose the Canada-minus-US difference in health-adjusted life expectancy at age 19. For the first counterfactual, death rates from the US were used to compute Canadian health-adjusted life expectancy assuming US mortality. For the second counterfactual, HUI3 score averages from the US were used to compute Canadian health-adjusted life expectancy assuming US morbidity.

The study was approved by the Institutional Review Board of Portland State University.

## Results

The overall response rates for Canada and the US were 65.5% and 50.2% [18,19]. Given that the JCUSH was conducted using random digit dialing, invalid telephone numbers created difficulties in the conduct of the survey and in the computation of the response rate. Given the small number of telephone companies in Canada, Statistics Canada was able to validate telephone numbers for working residential telephones selected for dialing in Canada, while for the US sample, Statistics Canada was unable to validate telephone numbers [18]. Using validated telephone numbers in Canada, an overall household response rate of 72% was obtained. One respondent was selected for each of the responding households, with an overall person-level response rate of 90.9%, yielding an overall response rate of 65.5%.

For the US, because of the large number of unvalidated telephone numbers, the response rate was calculated differently. In the US sample, the resolution rate represents the proportion of sample telephone numbers that could be positively identified as residential or nonresidential; the resolution rate was 80.4%. The majority of the unresolved telephone numbers reached persons or machines that hung up before identifying themselves or rang with no answer. The cooperation rate measures the proportion of known households within which an interview was completed; the cooperation rate was 62.4%. The overall response rate is the product of the resolution and cooperation rates, 50.2% [18].

Table 1 presents basic descriptive information on JCUSH respondents. Although the two populations are similar, obesity appears to be more prevalent in the US, while the proportion with more than a high school-level education is lower in Canada.

**Table 1:** Sociodemographic Characteristics of the Samples

	Canada (%)	United States (%)
**Age, Years**		
18-24	13.2	11.6
25-29	8.3	8.3
30-34	9.0	10.8
35-39	10.6	10.2
40-44	11.2	11.4
45-49	10.6	9.8
50-54	8.6	9.3
55-59	8.1	7.3
60-64	4.8	5.2
65-69	4.9	4.8
70-74	4.0	4.4
75-79	3.2	3.7
80-85	3.5	3.2
**Females**	50.9	52.0
**Education, Grade**		
<12	19.7	11.8
12+	80.3	88.2
**White** **Household income quintiles**	82.1	81.8
Lowest	17.0	15.3
Lower middle	17.5	16.4
Middle	16.1	14.5
Upper middle	17.6	15.2
Highest	16.0	14.8
Missing	15.9	23.9
**Body Mass Index**		
Underweight	2.8	2.2
Normal Weight	47.8	43.2
Overweight	34.0	33.9
Obese	15.3	20.7
**Current smoker**	24.9	22.4

*Note*. Weighted percentages

Source: Sanmartin, Ng, Blackwell, Gentleman, Martinez & Simile [19]

Table 2 compares the demographic characteristics of the US and Canadian JCUSH surveys to contemporary surveys of the non-institutionalized population in both countries: the 2003 US Behavioral Risk Factor Surveillance System Survey [44] and 2003 Canadian Community Health Survey [45]. (Comparisons between the JCUSH and census data are found in [27,46], and [47].) The age distributions in the JCUSH surveys match the age distributions in the contemporary population health surveys in both countries. In both countries, those who were married were overrepresented in the JCUSH, and those with less than a high school education were underrepresented.

**Table 2:** Comparison of JCUSH to 2003 US Behavioral Risk Factor Surveillance System Survey and 2003 Canadian Community Health Survey

	US JCUSH (18+)	US BRFSS (18+)	Canada JCUSH (20+)	Canada CCCHS (20+)
% Male	48.0	48.5	48.9	49.0
% < High School	11.8	12.3	19.8	19.2
% Never Married (Single)	19.2	19.5	18.7	20.2
% Legally Married (and not separated)	63.7	61.2	67.5	66.6
% Separated, Divorced, or Widowed	17.1	19.3	13.9	13.3
				
% Age < 45	52.3	51.6	50.6	49.9
% Age 45-64	31.7	31.5	33.2	33.8
% Age 65+	16.0	16.9	16.2	16.3

Sources: Centers for Disease Control, *Behavior Fisk Factor Surveillance System Survey 2003 *[44] and Statistics Canada, *Canadian Community Health Survey 2003 *[45].

The prevalence of a number of chronic conditions in the two countries based on results from the JCUSH is reported in Table 3. The prevalence of a number of chronic conditions appears to be higher in the US than in Canada.

**Table 3:** Comparison of Prevalence of Chronic Conditions, US and Canada, in % (18+)

Chronic Condition	US, JCUSH	Canada, JCUSH
Angina	2.9	3.7
Arthritis	18.7	16.8
Asthma	11.4	10.4
Depression	8.7	8.2
Diabetes	6.7	4.7
Emphysema or Chronic Obstructive Pulmonary Disease	3.6	2.2
Heart Attack, ever had	3.0	3.3
Heart Disease	6.0	5.1
Hypertension	22.7	18.3

Source: Sanmartin, Ng, Blackwell, Gentleman, Martinez & Simile [19].

Three different methods were used to compare the health of those residing in Canada and the US. First, Table 4 shows unadjusted results and adjusted results based on a linear regression model that included gender, race, education, income, smoking status, and BMI. For both unadjusted and adjusted results using established standards for interpreting HUI3 scores [48,49], mild morbidity burdens were observed for respondents 18 through 39 years regardless of country. For the 40+ group, Canadians appear to experience higher levels of HRQL than US residents. In the adjusted results, the differences in mean HUI3 scores for the 40-64 age groups and 65+ age groups are clearly quantitatively important (≥0.03), statistically significant for the 40-64 age groups, and almost statistically significant for the 65+ age groups. In the unadjusted results, the differences are smaller; for the 40-64 group, the difference of 0.01 is statistically significant and sufficiently large in the context of population health to be meaningful. Results of comparisons of health in the white-only Canadian and US populations, both unadjusted and adjusted, are similar to the results for comparisons of the entire sample.

**Table 4:** Unadjusted and Adjusted Mean Health Utilities Index Mark 3 Scores by Age and Country

Mean Unadjusted Health Utilities Index Mark 3 Scores by Age and Country				
**Age Range**	**Canada**	**United States**	**Canada-US**	**p-value**

18-39	0.91	0.91	0.00	0.59
40-64	0.88	0.86	0.02	<0.001
65+	0.79	0.78	0.01	0.37
Note: P-value is for test that the mean HUI3 scores in Canada and the US differ.				

**Mean Unadjusted Health Utilities Index Mark 3 Scores by Age and Country, White-Only**				

**Age Range**	**Canada**	**United States**	**Canada-US**	**p-value**

18-39	0.91	0.92	-0.01	0.42
40-64	0.88	0.87	0.01	0.06
65+	0.80	0.80	0.00	0.95
Note: P-value is for test that the mean HUI3 scores in Canada and the US differ.				

**Mean Adjusted Health Utilities Index Mark 3 Scores by Age and Country**				

**Age Range**	**Canada**	**United States**	**Canada-US**	**p-value**

18-39	0.91	0.91	0.00	0.66
40-64	0.89	0.86	0.03	<0.001
65+	0.82	0.79	0.03	0.06
Note. Overall HUI3 scores are adjusted for gender, race, education, income, smoking status, and BMI. P-value is for test that the mean HUI3 scores in Canada and the US differ.				

**Mean Adjusted Health Utilities Index Mark 3 Scores by Age and Country, White-Only**				

**Age Range**	**Canada**	**United States**	**Canada-US**	**p-value**

18-39	0.92	0.92	0.00	0.92
40-64	0.91	0.87	0.04	<0.001
65+	0.83	0.80	0.02	0.20

Note. Overall HUI3 scores are adjusted for gender, race, education, income, smoking status, and BMI. P-value is for test that the mean HUI3 scores in Canada and the US differ.

A comparison of the distribution of levels within each of the eight HUI3 attributes between Canada and the US (data not shown) reveals that the prevalence of moderate and severe disability is systematically higher in the US than in Canada.

Second, mortality rates are lower in Canada [4,50-54]. The effects of the differences in mortality rates between the countries are reflected in the estimates of the additional life expectancy of 19-year-olds (Table 5). Life expectancy at birth is also higher (and the infant mortality rate is lower) in Canada than in the US.

**Table 5:** Comparisons of Health, Poverty, and Income Inequality in Canada and the United States

	Canada	United States	Canada - US
Life Expectancy at Birth in years^a^	79.7	77.2	2.5
Infant Mortality Rate, deaths per 1,000 live births^a^	5.4	7	-1.6
Life Expectancy at Age 19 in years	60.6^b^	58.3^b^	2.3
Poverty rate^c ^in %	12	17	-5
Poverty rate^c ^among the elderly in %	6	23	-17
Gini coefficient^e^	0.32	0.38	-0.06
Health-adjusted Life Expectancy (HALE)in years^f^	52.0	49.3	2.7
Counterfactual #1, HALE in Canada with Canadian morbidity and US mortality	49.9	HALE Canada actual - #1 = 1.8	
Counterfactual #2, HALE in Canada with US morbidity and Canadian mortality	50.8	HALE Canada actual - #2 = 0.9	

^a^Estimates for 2002 [2]

^b^Based on 2003 Life Tables for Canada [40,41] and 2003 Life Tables for the US [83].

^c^Based on Organization for Economic Co-Operation and Development estimates 2008; [55]; OECD average 11%.

^d^Based on Organization for Economic Co-Operation and Development estimates 2008; [55].

^e^Organization for Economic Co-Operation and Development estimates 2008; [55]; OECD average 0.31.

^f^Based on mean HUI3 score by age category observed in JCUSH and life tables for 2002-2003 for Canada and the United States. The difference between the US and Canadian estimates of HALE is significant at p < 0.0001.

Third, HALE estimates reflect differences in both morbidity and mortality (Table 5), with a difference of 2.7 more years of "perfect health" in Canada compared to the US. The 95% confidence interval around the estimate of HALE for Canada is 51.5 to 52.5 years; for the US, the 95% confidence interval is 48.9 to 49.7 years. The difference between the estimates of HALE for Canada and the US is statistically significant with p < 0.0001. The 95% confidence interval around the estimated difference of 2.7 years is 2.0 to 3.4.

Counterfactual #1 shows what the HALE in Canada would have been if Canada had experienced US mortality rates and Canadian morbidity; it differs from the actual estimate by 1.8 years of perfect health. Counterfactual #2 shows what HALE in Canada would have been if Canada had Canadian mortality rates but US morbidity. The difference between the actual and the counterfactual is 0.9 years of perfect health. Thus, differences both in mortality and in morbidity are important in accounting for the higher HALE in Canada. The results also indicate that mortality differences are quantitatively more important than morbidity differences in accounting for the difference in HALE.

Given that differences in poverty and social and economic inequality may be important in accounting for differences in HALE in Canada and the US, Table 5 includes data on poverty rates and income inequality based on data from the Organisation for Economic Co-operation and Development 2008 [55]. The poverty rate is defined as the proportion of individuals with incomes below 50% of the median. Income inequality is measured using the Gini coefficient that reflects the difference between the actual distribution of household income and an ideal distribution of complete equality. Gini coefficients range from 0.00 (complete inequality) to 1.00 (complete equality). Clearly, poverty is more prevalent in the US than in Canada, especially among the elderly. Further, income inequality is substantially higher in the US than in Canada.

## Discussion

Why are HRQL, life expectancy, and HALE apparently higher in Canada? The JCUSH is a cross-sectional survey, so caution must be exercised in interpreting the Canada-US comparisons. There are two, not mutually exclusive, categories of potential explanations for the differences observed: differences in access to health care and differences in poverty and inequality.

### Access to Health care

It is notable that differences in health between the US and Canada are evident for the 40+ group. Variability in health insurance coverage across the life cycle in the US as compared to the universal "prenatal-to-grave" coverage in Canada is one potential explanation for the difference in HRQL. Mojtabuui and Olfson [56] note that for depression, the severity of symptoms is more closely related to treatment-seeking in Canada than in the US, providing indirect evidence of the effects of universal access. For the period 1997-98 through 2002-03, Nolte and McKee [53] note that deaths for conditions regarded as amenable to treatment declined much more rapidly in 18 other industrialized countries, including Canada, than in the US, evidence consistent with the importance of universal access. Similarly, James et al. [57] examine avoidable mortality in Canada during the 25-year period after the introduction of universal health insurance and note a steady decline in disparities in mortality among socioeconomic groups over that time period.

A number of studies in the US have examined the effects of the lack of health insurance on health. Clearly, there are major methodological challenges in controlling for the fact that not having insurance is not a random event. Using data from the 1971-1975 and the 1987 follow-up National Health and Nutrition Examination Surveys, Franks et al. [58] estimate that, controlling for a wide variety of determinants of health, the hazard rate for lacking insurance is 1.25 (95% confidence interval 1.00 to 1.55). Using an instrumental variables approach and data from the Health and Retirement Survey for subjects 55-61 years old in 1991, Hadley and Waidman [59] found that those with continuous insurance coverage were less likely to die and more likely to be healthy, controlling for a wide variety of determinants of health. Wilper et al. [60] also found that, controlling for a wide variety of determinants of health, being uninsured increased the risk of mortality; see also [61]. Further, Levy and Meltzer [62] note that there is strong evidence that health insurance improves health status in vulnerable populations.

### Poverty and Inequality

Data in Table 5 indicate that rates of poverty, especially among the elderly who are, in general, at an elevated risk for both mortality and morbidity, are lower in Canada than in the US. Further, income inequality is substantially higher in the US than in Canada. Several investigators, including Smith 1999 [63], have provided evidence that the relationship between health and income (or wealth) is concave, with the effects strongest at low incomes and weakest at high incomes [64]. Thus, lower rates of poverty in Canada could account for at least some of the gap in HALE.

Controlling for the level of income and the determinants of health, mixed results have been reported in investigations of the associations between income inequality and mortality [64-70]. In international comparisons, there is, in general, little evidence of an effect of inequality on mortality. However, the US experience represents an important exception. Several studies found a relationship between income inequality and working-age mortality for US metropolitan areas but not for Canadian metropolitan areas [4,71]. Ross et al [4] also found a relationship between increased income segregation and increased mortality in US metropolitan areas, but again not in Canadian metropolitan areas. Further, in a number of US studies when the proportion of the population that is African American is included in the analysis, the effects of inequality often becomes statistically insignificant [65,70]. When morbidity, disability, or overall HRQL are used as the measure of health instead of mortality, there is evidence of a relationship between inequality and health [67-69].

The result that the proportion of a state's population that is African American is important in explaining state-level variations in mortality [16,17,65,70] suggests that the legacies of slavery and racial discrimination may be important in accounting for the HALE gap. Yet comparisons of overall HRQL between the white-only Canadian and US populations (Table 4) suggest that race does not come close to accounting fully for the gap. (See also [72].) Torrey and Haub [50] compare mortality rates for the Canadian and US populations. The results that they report suggest that Canadians, white-only and overall, experience lower mortality risks than in the US, especially for those under 65 years of age.

### Access, Poverty, and Inequality

The health disparity apparent between the US and Canada for the mid-life and older groups could be associated with the delayed effects of childhood health on adult health (latency model) and/or the cumulative effects (life-course or pathways model) of more restricted access to health care and higher levels of social and economic inequality in US relative to Canada [1,3,4,46,47,50-52,70,73-82]. The latency model postulates that discrete events early in life (e.g., birthweight) substantially affect lifetime health [74,75,79]. The life-course model suggests that health trajectories result from the cumulative effects of risk and protective factors [74,75,79]. Both models imply that by middle age, the effects of access to health care and social and economic inequality over the life course become manifest with the onset of chronic conditions and health impairments. In Canada, relatively generous redistributive tax policies (transfers from upper- to lower-income groups) and greater public-sector investments in education, community recreation, and public transit (transfers in kind), have ameliorated some of the consequences of social hierarchy. While there is a socioeconomic gradient in health status in Canada, it is less dramatic than in the US [46,47,72].

In 2002, life expectancy at birth in Canada exceeded that in the US by 2.5 years. In the US, life expectancy at birth in 1979-81, 1989-91, and 2003 was 73.9, 75.4, and 77.5 years respectively [83]. Thus, the difference between US and Canadian life expectancy at birth in 2002 exceeded the gain in US life expectancy at birth experienced over the 1989-91 to 2002 period. Similarly, the US life expectancy for a 20-year-old in 1979-81, 1989-91, and 2003, was 55.5, 56.6, and 58.4 years, respectively [83]. Again, the difference between the US and Canada in additional life expectancy at age 19 (or 20) in 2002 exceeds the gain for US 20 year-olds over the previous decade. The differences in life expectancy observed between the US and Canada are quantitatively important.

This conclusion is reinforced by comparing HALE in Canada and the US. A 19-year-old Canadian can expect to experience 2.7 more years of perfect health than her/his US counterpart over their lifetimes. In 2001 in Canada, the difference in HALE at birth for males and females in the top one-third of the income distribution as compared to the bottom one-third was 4.7 and 3.2 years, respectively [84]. The difference in HALE between the US and Canada of 2.7 is more than half of the gap between the HALE for highest- and lowest-income groups observed in Canada. Recently published estimates of HALE by income decile for Canada provide additional insights [38]. The difference in HALE for males between the lowest quintile and the second (next lowest) quintile is 4.35 years; the difference between the second and third quintiles is 2.30 years. For females, the corresponding results are 3.55 and 1.75 years. The Canada-US difference in HALE is quantitatively important.

Our HALE estimate of 49.3 for a 19-year-old person in the US is very similar to the one reported by Muennig et al. [6] of 51.1 for persons 18 years of age in the US. Muennig et al. [6] used EQ-5D [85] scores from the 2000 Medical Expenditure Panel Survey to estimate HALE. The difference between our and their estimate is likely due to the fact that EQ-5D may underestimate the burden associated with mild disability and that their estimate is for persons age 18 while our estimate is for persons age 19. Similarly, using measures of healthy days lost from the Behavioral Risk Factor Surveillance System Survey and EQ-5D, Jia and Lubetkin [9] provide an estimate of quality-adjusted life expectancy for an 18-year-old in the US as 52.0 years.

A limitation of the JCUSH is the low response rates and that the US response rate was lower than the Canadian. It is unclear how the differential response rate might affect comparisons of health in Canada and the US. If nonresponse is more likely among those with lower health status, it is possible that results based on the JCUSH understate the difference between Canada and the US. For both Canada and the US, married subjects are slightly over-represented and those with less than a high school education are slightly under-represented in the JCUSH, implying that population health in both countries among the non-institutionalized population may be over-estimated. It is possible that the Canada-US gap is understated.

Even assuming that age-specific mortality rates are estimated without error, there is some imprecision in our estimates of HALE for each country. McIntosh et al. [38] report confidence intervals on estimates of remaining HALE in Canada at age 25 based on HUI3 scores from the 2000-2001 Canadian Community Health Survey. The estimate of HALE for males was 47.3 years, with a 95% confidence interval of 46.9 to 47.8; for females, the results were 53.2, with a 95% confidence interval of 52.9 to 53.5 (personal communication from Phillippe Fines, November 23, 2009). The confidence intervals for our estimates of HALE are not as narrow as those reported by McIntosh et al. Nonetheless, the limited sample size in the JCUSH is sufficient to provide reasonable precision. Further, it is unlikely that HALE in Canada and the US overlap.

Another potential limitation of the analyses reported here is the use of a multi-attribute utility function estimated using preference scores obtained from a random sample of residents of Canada to value health states observed in the US. It is possible that the preferences for health states differ between Canada and the US. We are unaware of any direct evidence on this issue. Nonetheless, it is important to note that the parameter values of HUI3 scoring functions estimated in other countries, including France [86], Spain [87], and the Netherlands [88], are very similar to the parameter values for the Canadian HUI3 scoring function [21].

The results reported in this paper have implications for future research. The Joint Survey is valuable in that it provides a comprehensive and direct comparison of the health in the US and Canada. Yet distinguishing among the potential explanations for the differences in health between the two countries would require longitudinal data. Perhaps it is time for Canada and the US to contemplate a joint longitudinal survey.

## Conclusions

In conclusion, population health, HRQL, life expectancy, and HALE in Canada compare favorably to the United States. The difference in health between the two countries seems to be associated with substantial differences in access to care as well as substantial differences in social and economic inequality. The results of the Canada-US comparisons have implications for health care and social policy in the United States [78].

## Competing interests

It should be noted that DF has a proprietary interest in Health Utilities Incorporated (HUInc.), Dundas, Ontario, Canada. HUInc. distributes copyrighted Health Utilities Index (HUI) materials and provides methodological advice on the use of HUI. It should be noted that HUInc. received no payment for the use of HUI in the survey discussed in this manuscript. None of the other authors declare any competing interests.

## Authors' contributions

DF conceived of the project and was actively involved in its design, the design of the analyses and interpretation of the results, and responsible for drafting the paper. MSK participated actively in the design of the project and the analyses and the interpretation of the results and reviewed manuscript drafts. NH participated actively in the design of the project, conducted the analyses, and was involved in the interpretation of the results; she reviewed drafts of the manuscript. BHM participated actively in the design of the project and the analyses and the interpretation of the results; he reviewed drafts of the manuscript. All authors have read and approved the final manuscript.
